# Sex difference in inappropriate therapy and survival among 1471 implantable cardioverter‐defibrillator recipients

**DOI:** 10.1111/jce.14003

**Published:** 2019-06-18

**Authors:** Achim Leo Burger, Herwig Schmidinger, Robin Ristl, Thomas Pezawas

**Affiliations:** ^1^ Division of Cardiology, Department of Internal Medicine II Medical University of Vienna Vienna Austria; ^2^ Center for Medical Statistics, Informatics, and Intelligent Systems, Section for Medical Statistics Medical University of Vienna Vienna Austria

**Keywords:** sex, implantable cardioverter‐defibrillator, inappropriate therapy, sex difference

## Abstract

**Introduction:**

To assess a potential relationship between sex and outcome in recipients of an implantable cardioverter‐defibrillator (ICD).

**Methods and Results:**

All 1471 ICD recipients between 2000 and 2015 were sex‐related analyzed with the following outcome parameters: overall survival (OS), the occurrence of inappropriate and appropriate antitachycardia pacing (ATP), and shock therapy. We followed 1206 (82%) male and 265 (18%) female ICD recipients during 4.1 ± 3.6 and 4.3 ± 3.8 years, respectively, (*P* = .369). Kaplan‐Meier analysis revealed that there was no significant difference in OS between female and male patients (*P* = .132). After adjustment for relevant confounding factors in a multivariate model, sex remained a nonsignificant predictor of overall mortality (hazard ratio [male] = 1.11; *P* = .493). Negative binomial regression analysis revealed that women received less appropriate ATP therapy (rate ratio [RR] = 0.37; *P* = .043), whereas rates of appropriate shock therapy (RR = 1.95; *P* = .369) did not differ between women and men. No significant differences were observed in the occurrence of inappropriate ATP (RR = 1.22; *P* = .715) and inappropriate shock therapy (RR = 0.64; *P* = .121).

**Conclusion:**

Female and male patients equally benefit from ICD therapy in terms of OS. Women are less likely to receive appropriate ATP therapy, whereas appropriate shock and inappropriate ATP and shock therapy are independent of sex.

## 1 INTRODUCTION

Implantable cardioverter‐defibrillator (ICD) therapy is associated with a significant reduction in overall mortality in patients at high risk for ventricular arrhythmias.^1^, ^2^, ^3^, ^4^, ^5^ Previous data indicated that the outcome of ICD therapy is different between female and male patients.^6^, ^7^, ^8^ However, it is still controversial to what extent the outcome of ICD therapy is influenced by sex and if this should impact on programming strategies. Recent studies from European registries reported that females have lower mortality rates compared to men.^6^, ^7^, ^8^ In contrast to these results, data from an Israeli registry^9^ and a North‐American registry^10^ could not identify a difference in overall survival (OS). The present large‐scale study aims to assess sex‐related differences in the outcome of ICD therapy in an unselected real‐world population and its possible impact on treatment strategies.

## 2 METHODS

### 2.1 Design and study population

This is a retrospective, longitudinal study of consecutive ICD recipients at the Department of Cardiology, Medical University of Vienna. The analysis included all patients who received an ICD (VVI or DDD) or a cardiac resynchronization therapy‐defibrillator (CRT‐D) device between January 2000 and May 2015 regardless of comorbidities, etiology, and reason for primary or secondary prevention. Indication for ICD implantation and device programming followed eligible guidelines at the time of implantation. This study was approved by the local ethics committee.

### 2.2 Data source

The data source for the underlying study was the database of the Arrhythmia Outpatient Department of the Medical University of Vienna. It is used for daily routine and provided all relevant data that was required for the underlying study, including accurate information on occurrence and classification of ICD therapy and patient's mortality. Follow‐up was conducted at least every 6 months or after a suspected arrhythmia at the arrhythmia outpatient department and included device interrogation for analysis of delivered therapy. At least two specialized cardiologists reviewed the device interrogation and classified the episodes in appropriate or inappropriate by agreement. This classification was based on stored electrograms, including stability, onset, and morphology of the arrhythmic event. An episode was classified as inappropriate, if ICD therapy was delivered for other reasons than ventricular tachycardia or ventricular fibrillation. This included supraventricular tachyarrhythmias, atrial fibrillation and atrial flutter. In case of disagreement between the two cardiologists, a third specialized cardiologist was included and reviewed device interrogations. The duration of follow‐up was calculated from the time of ICD implantation until the last device interrogation.

### 2.3 Outcome parameters

The primary endpoint was time to antitachycardia pacing (ATP) or shock therapy. All‐cause mortality was the secondary endpoint. In case of death, physicians and family members, as well as witnesses, were interviewed for detailed circumstances.

### 2.4 Statistical analysis

The statistical analysis was conducted with the software program SPSS (version 25.0, SPSS Inc, Chicago, IL) with a significance level of a two‐sided *P* ≤ .05. Categorical variables are presented as number and percentage, continuous variables as mean ± standard deviation. Rate ratios (RRs) were calculated to compare counts of inappropriate and appropriate therapy applying negative binomial regression analysis. The individual follow‐up time of each patient was accounted for in terms of an offset variable in the model. Robust variance estimation was used in the calculation of confidence intervals and Wald test *P* values for RRs. Kaplan‐Meier analysis and the logrank test were used to evaluate differences in time to the first occurrence of inappropriate therapy and OS. Univariate and multivariate Cox regression analysis was performed to determine predictors of overall mortality and for one or more than one inappropriate shock therapies. The univariate model was fit for age, implantation for primary preventive indication, underlying heart disease, treatment with antiarrhythmic drugs class 3, left ventricular ejection fraction (LVEF; divided into normal, mild, moderate, and severe reduction) and device type (VVI, DDD, and CRT‐D). Variables with a *P* < .10 were included in the multivariate analysis.

## 3 RESULTS

The study enrolled a total of 1471 ICD recipients: 265 (18%) were female and 1206 (82%) were male patients. Baseline clinical characteristics are presented in Table 1. Follow‐up time did not differ between the two groups. Female patients suffered less often from ischemic heart disease, but more often from hypertrophic cardiomyopathy, channelopathies and “other” rare conditions such as postmyocarditis, congenital heart disease, and severe valvular heart disease. Women were less often treated with antiarrhythmic drugs class 3 and had a better left ventricular function. Females were more often implanted with a dual chamber ICD (DDD) and had less frequent hypertension and hyperlipidemia (details see Table 1).

**Table 1:** Baseline clinical characteristics stratified according to sex

	Female	Male	*P* value
Number of patients (n/phase)	265	1206	
Age (mean ± SD)	59.6 ± 16.2	61.7 ± 13.2	0.059
Follow‐up (mean ± SD), y	4.3 ± 3.8	4.1 ± 3.6	0.369
Ischemic Heart Disease (n,%)	119 (44.9)	789 (65.4)	<0.001
Dilative Cardiomyopathy (n,%)	56 (21.1)	249 (20.6)	0.870
Hypertrophic Cardiomyopathy (n,%)	23 (8.7)	50 (4.1)	0.002
Channelopathies (n,%)	29 (10.9)	36 (3.0)	<0.001
Idiopathic VF (n,%)	10 (3.8)	22 (1.8)	0.049
Others (n, %)	28 (10.6)	60 (5.0)	0.001
Antiarrhythmic drugs according to Vaughan Williams			
Class 1 (n,%)	3 (1.1)	9 (7.5)	0.971
Class 2 (n,%)	205 (77.4)	988 (81.9)	0.187
Class 3 (n,%)	72 (27.2)	415 (34.4)	0.028
Class 4 (n,%)	32 (12.1)	107 (8.9)	0.835
Sotalol (n,%)	10 (3.8)	44 (3.6)	0.509
ACE Inhibitors/ARB (n,%)	187 (70.6)	947 (78.5)	0.143
Digitalis glycosides (n,%)	25 (9.4)	143 (11.9)	0.116
Aldosterone Antagonists (n,%)	109 (41.1)	477 (39.6)	0.313
Diuretics (n,%)	139 (52.5)	677 (56.1)	0.182
LVEF (existing data, %)			
LVEF ‐ normal (n, %)	71 (26.8)	163 (13.5)	<0.001
LVEF ‐ mild reduction (n, %)	31 (11.7)	127 (10.5)	0.401
LVEF ‐ moderate reduction (n, %)	31 (11.7)	217 (18.0)	0.028
LVEF ‐ severe reduction (n, %)	99 (37.4)	607 (50.3)	0.001
Primary prevention (n, %)	125 (47.2)	623 (51.7)	0.149
VVI (n, %)	105 (39.6)	524 (43.4)	0.210
DDD (n, %)	104 (39.2)	382 (31.7)	0.018
CRT‐D (n, %)	57 (21.5)	299 (24.8)	0.270
Hypertension (n,%)	155 (58.5)	799 (66.2)	0.017
Hyperlipedimia (n,%)	66 (24.9)	389 (32.2)	0.019
Diabetes Mellitus (n,%)	44 (16.6)	260 (21.6)	0.071

Abbreviations: AAR, antiarrhythmic drugs; ACE, angiotensin converting enzyme; ARB, angiotensin receptor blockers; CRT‐D, cardiac resynchronization therapy‐defibrillator; DDD, dual chamber implantable cardioverter‐defibrillator; ICD, implantable cardioverter‐defibrillator; LVEF, left ventricular ejection fraction; SD, standard deviation; VF, ventricular fibrillation.

### 3.1 Overall survival

**Figure 1:**
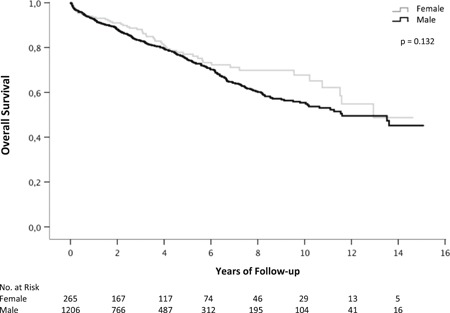
Kaplan‐Meier curves analyzing overall survival stratified for sex

Kaplan‐Meier analysis showed no significant difference in OS between female and male patients (*P* = .132; Figure 1). After 5 and 10 years of follow‐up, the probability for OS was 77.0% and 67.7% in female patients and 74.6% and 55.4% in male patients, respectively. Univariate and multivariate Cox regression analysis was performed to determine predictors for overall mortality and to account for potential confounding factors. In the multivariate analysis, age (hazard ratio [HR] = 1.05; 95% confidence interval [CI], 1.04‐1.06; *P* = <.001), treatment with antiarrhythmic drugs class 3 (HR = 1.30; 95% CI, 1.05‐1.62; *P* = .017), normal LVEF (HR = 0.65; 95% CI, 0.43‐0.99; *P* = .045), severely reduced LVEF (HR = 1.43; 95% CI, 1.13‐1.82; *P* = .003), channelopathies (HR = 0.47; 95% CI, 0.17‐1.30; *P* = .145), and idiopathic ventricular fibrillation (HR = 0.31; 95%CI, 0.04‐2.20; *P* = .241) were relevant confounding variables for OS. In an adjusted model for these confounding factors, sex was not associated with OS (HR [male] = 1.11; 95% CI, 0.82‐1.51; *P* = .493). Ischemic heart disease was a nonsignificant predictor for OS in the multivariate analysis (HR = 0.90; 95%CI, 0.70‐1.15; *P* = .382). Furthermore, Kaplan‐Meier analysis revealed no significant difference between sex in OS in patients stratified according to the prevention strategy: primary prevention (*P* = .403) and secondary prevention (*P* = .137).

### 3.2 First inappropriate therapy

**Figure 2:**
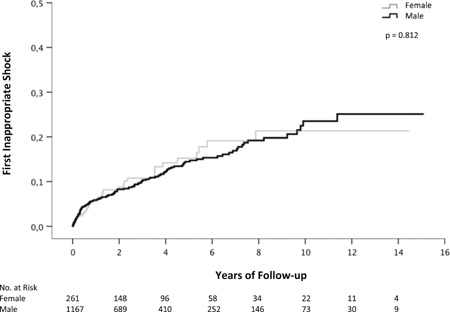
Kaplan‐Meier curves showing the association between the first occurrence of inappropriate shock therapy and sex

Kaplan‐Meier analysis was performed to analyze the time until the first occurrence of inappropriate ATP and shock therapy. There was no significant difference in first inappropriate ATP (*P* = .468) and first inappropriate shock therapy (*P* = .812; Figure 2) between female and male patients. After 5 and 10 years of follow‐up, the probability for first inappropriate shock therapy was 15.2% and 21.3% in female patients and 14.5% and 23.5% in male patients, respectively. Uni‐ and multivariate Cox regression models were calculated to analyze the risk of receiving one or more than one inappropriate shock therapies. In the multivariate analysis, age (HR = 0.99; 95%CI, 0.98‐1.00; *P* = .127), primary preventive implantation (HR = 0.78; 95% CI, 0.56‐1.10; *P* = .152), moderately reduced LVEF (HR = 0.65; 95% CI, 0.41‐1.02; *P* = .061) and implantation with a CRT‐D (HR = 0.44; 95% CI, 0.23‐0.84; *P* = .013) were relevant confounding variables. After adjustment for these confounding factors, sex (HR (male) = 1.05; 95% CI, 0.69‐1.59; *P* = .829) was not associated with the risk for one or more than one inappropriate shocks.

### 3.3 Incidence of inappropriate therapy

A total of 1783 events of inappropriate ATP and 968 events of inappropriate shock therapy occurred in the overall study population. Rate per patient year for inappropriate ATP and shock therapy ranged between 0.11 and 0.36 and did not differ significantly (Table 2). Negative binomial regression analysis did not reveal a significant difference between female and male patients in inappropriate ATP (RR = 1.22; 95% CI, 0.42‐3.59; *P* = .715) and in inappropriate shock therapy (RR = 0.64; 95% CI, 0.36‐1.13; *P* = .121).

**Table 2:** Number of ICD ATP and shock therapies and rates per patient year stratified according to sex

	Number of events (rate per patient year)		Female vs male	
	Female	Male	RR (95% CI)	*P* value
Inappropriate ATP	405 (0.36)	1378 (0.28)	1.22 (0.42‐3.59)	0.715
Inappropriate shock	120 (0.11)	848 (0.17)	0.64 (0.36‐1.13)	0.121
Appropriate ATP	1116 (0.98)	11849 (2.42)	0.37 (0.14‐0.97)	0.043
Appropriate shock	526 (0.46)	1647 (0.34)	1.95 (0.46‐8.32)	0.369

*Note*: Negative binomial regression analysis of inappropriate and appropriate therapies.

Abbreviations: ATP, antitachycardia pacing; CI, confidence interval; ICD, implantable cardioverter‐defibrillator; RR, rate ratio.

### 3.4 Incidence of appropriate therapy

A total of 12 965 events of appropriate ATP and 2173 events of appropriate shock therapy occurred in the overall study population. Rate per patient year of appropriate ATP was 0.98 in female patients, compared with 2.42 in male patients (Table 2). Negative binomial regression analysis revealed a lower incidence of appropriate ATP in female patients compared with male patients (RR = 0.37; 95% CI, 0.14‐0.97; *P* = .043). The analysis did not reveal a significant difference in the occurrence of appropriate shock therapy (RR = 1.95; 95% CI, 0.46‐8.32; *P* = .369).

An additional negative binomial regression analysis showed that patients with channelopathies had a significantly lower rate of appropriate ATP (RR = 0.23; 95% CI, 0.06‐0.87; *P* = .031) compared with patients without channelopathies. The analysis did not reveal a significant difference in inappropriate shock therapy associated with channelopathies (RR = 0.73; 95% CI, 0.33‐1.61; *P* = .434).

## 4 DISCUSSION

The main findings of the underlying study are that (a) female and male patients equally benefit from ICD therapy, (b) OS and inappropriate therapy are independent of sex, and (c) women receive less appropriate ATP therapies.

The literature on sex‐related differences in ICD therapy is incomplete. There are studies from European registries^6^, ^7^, ^8^ and a meta‐analysis by Conen et al^11^ that reported reduced overall mortality in female ICD recipients. Median follow‐up times in these studies ranged between 2.7 and 3.3 years.^6^, ^7^, ^8^ However, these studies included patients with a primary preventive ICD indication only. In contrast, our analysis includes a comprehensive study population with primary and secondary preventive ICD recipients with ischemic and nonischemic cardiomyopathies. We have a mean follow‐up of 4.1 years with a maximum of 15.1 years, which is considerably longer compared with previous trials. The results of the present study add important information to the existing body of literature. We demonstrate in a large cohort that mortality rates among ICD recipients do not differ between female and male patients. To the best of our knowledge, there are only two studies available which did not find any sex‐related difference: a large North‐American registry^10^ did not identify a significant difference in overall mortality between sex in patients with primary and secondary ICD indication. This result was confirmed by an Israeli registry that also included primary and secondary preventive ICD recipients.^9^ Patients were followed for 1 year in the North‐American registry^10^ and for a median of 323 days in the Israeli registry.^9^ Thus, the present study provides new and valuable long‐term data in this context.

Inappropriate ATP and shock therapy represent an anticipated risk in ICD therapy. Previous research showed that inappropriate therapy is associated with worse clinical outcome and increased overall mortality.^12^, ^13^ Optimized ICD programming strategies and discrimination algorithms are crucial to reduce inappropriate therapy burden in primary and secondary prevention.^14^, ^15^, ^16^, ^17^ Sex‐specific differences in the occurrence of inappropriate therapy were reported by Tomkins et al^18^ in a substudy of the randomized trials MADIT II and MADIT‐CRT. In contrast, other studies^6^, ^7^, ^8^, ^10^ did not report an influence of sex on the risk of inappropriate therapy. In addition, a meta‐analysis by Conen et al^11^ did not identify a significant difference in the risk of inappropriate therapy between female and male patients with a pooled HR of 0.99 (95% CI, 0.56‐1.73; *P* = .927). The underlying study adds to the existing literature that women and men have an equal risk for inappropriate ATP and shock therapy during long‐term follow‐up. After 10 years of follow‐up, the risk of first inappropriate shock was 21.3% in females and 23.5% in males (*P* = .812).

Conflicting results were reported regarding appropriate therapy. Some studies indicated that women receive less appropriate ATP and shock therapies. A North‐American registry^10^ reported a lower risk of appropriate ATP (HR = 0.73; *P* = .003) and appropriate shock therapy (HR = 0.69; *P* = .015) in females after a median follow‐up of 1 year. Similar results were reported from a French^6^ and a combined European registry^7^ after median follow‐up times of 2.8 and 2.4 years, respectively. A Dutch cohort^8^ showed a nonsignificant trend (HR = 0.81; *P* = .07) towards less appropriate therapy in women, after 3.3 years. Contrary to these findings, results from an Israeli registry^9^ showed no difference in appropriate therapy (HR = 1.17; *P* = .69) between men and women after 1 year of follow‐up. In the present study, after an average follow‐up of 4.1 years, rates of appropriate ATP therapy (RR = 0.37; *P* = .043) were significantly lower in a woman compared with men. However, the occurrence of appropriate shock therapy (RR = 1.95; *P* = .369) was similar in female and male patients, suggesting an equal benefit of ICD implantation in our cohort. The lower rates of appropriate ATP in female patients may be explained by a larger proportion of channelopathies in women in this study cohort. Negative binomial regression analysis demonstrated that patients with channelopathies have a significantly lower incidence of appropriate ATP therapy, whereas the incidence of appropriate shock therapy is similar in patients with or without channelopathies.

LVEF is still the gold standard for selection of patients at risk for life‐threatening ventricular arrhythmias. ICD recipients with mild to moderate reduced LVEF might be particularly affected, because even those patients are at risk for arrhythmias during long‐term follow‐up.^19^, ^20^ In the underlying study, we did not observe sex‐related differences in regards to LVEF.

In this unselected study cohort, females represent the minority of ICD recipients with only 18% of all patients implanted between 2000 and 2015. This is in line with data from registries that report a proportion of female patients ranging between 15.1% and 21.2%.^6^, ^7^, ^8^, ^9^ Likewise, in major ICD landmark studies, women account for the minority of the study population.^3^, ^4^, ^21^, ^22^

### 4.1 Study limitations

This is an observational, single‐center study with a long follow‐up to analyze sex differences in ICD outcome. However, the retrospective and longitudinal design of the study accounts for certain limitations. Patients were included between 2000 and 2015 and evolving guidelines and ICD development may have influenced results. In addition to this, a control group without ICD implantation would have been necessary for a definite answer on the degree of ICD benefit in both sexes.

## 5 CONCLUSION

The underlying study demonstrates that female and male patients equally benefit from ICD therapy. OS and rates of appropriate shock therapy are similar in both groups, whereas rates of appropriate ATP are lower in female patients. Women and men have the same risk for inappropriate therapy.

## CONFLICT OF INTERESTS

The authors declare that there are no conflict of interests.
